# Final report on plasma ctDNA T790M monitoring during EGFR-TKI treatment in patients with EGFR mutant non-small cell lung cancer (JP-CLEAR trial)

**DOI:** 10.1093/jjco/hyac032

**Published:** 2022-03-22

**Authors:** Go Naka, Takuma Yokoyama, Kazuhiro Usui, Hiroo Ishida, Kazuma Kishi, Kohei Uemura, Yasuo Ohashi, Hideo Kunitoh

**Affiliations:** 1 National Center for Global Health and Medicine, Tokyo, Japan; 2 Kyorin University Hospital, Mitaka, Tokyo, Japan; 3 NTT Medical Center, Tokyo, Japan; 4 Showa University Northern Yokohama Hospital, Yokohama, Kanagawa, Japan; 5 Toho University Omori Medical Center, Tokyo, Japan; 6 The University of Tokyo, Tokyo, Japan; 7 Chuo University, Tokyo, Japan; 8 Japanese Red Cross Medical Center, Tokyo, Japan

**Keywords:** lung neoplasm, epidermal growth factor, mutation, liquid biopsy

## Abstract

Osimertinib is active against T790M-positive epidermal growth factor receptor mutant non-small cell lung cancer. We enrolled 122 sensitive epidermal growth factor receptor mutant non-small cell lung cancer patients who were planned to receive or were receiving first-/second-generation epidermal growth factor receptor tyrosine kinase inhibitors without disease progression and monitored plasma T790M every 1–2 months using the cobas® EGFR Mutation Test v2. We previously reported the concordance between T790M status in plasma and tissue. This is the final report on the sensitivity of plasma T790M and the efficacy of sequential osimertinib. The sensitivity was 21.1% (95% confidence interval: 6.1–45.6%). The best overall response was 25.0% (95% confidence interval: 9.8–46.7) in the plasma T790M-positive group and 28.6% (95% confidence interval: 8.4–58.1) in the plasma T790M-negative but tissue T790M-positive group. Median progression-free survival was 7.9 months (95% confidence interval: 4.7–17.5) for the former and 4.4 months (95% confidence interval: 3.0–N.E.) for the latter, with no statistically significant difference (*P* = 0.74).

## Teaser

In our study, with regular monitoring of plasma circulating tumor DNA (ctDNA) T790 in daily practice, there was no statistically significant difference in progression-free survival between the plasma ctDNA-positive and ctDNA-negative groups.

## Introduction

Treatment with first- or second-generation epidermal growth factor (EGFR)-tyrosine kinase inhibitors (TKIs) is effective for patients with non-small cell lung cancer (NSCLC) who harbor a sensitizing EGFR mutation. However, acquired resistance is inevitable after 9–14 months (1–3), and the most common mechanism of resistance is the EGFR T790M mutation, which accounts for approximately 60% of cases (4). Osimertinib, a third-generation EGFR-TKI, is reported to be highly active against T790M-positive NSCLC (5). To detect the T790M mutation, tumor re-biopsy is necessary. However, re-biopsies are often infeasible during standard care of NSCLC patients. Circulating tumor DNA (ctDNA) detected in plasma is recognized as a noninvasive biomarker for the molecular analysis of NSCLC. The cobas® EGFR Mutation Test (Roche Diagnostics K.K., Switzerland) is a companion diagnostic test for the detection of EGFR mutations in plasma specimens and has been approved to identify such patients with NSCLC (6). We conducted an observational study of plasma ctDNA in NSCLC patients with EGFR mutations receiving EGFR-TKIs and reported the monitoring results from baseline to the initiation of osimertinib (7). In this article, we present the final report of this study, including treatment results with osimertinib.

## Patients and methods

### Study population

Key eligibility criteria were as follows: (i) histologically and/or cytologically confirmed advanced or post-operative recurrent NSCLC harboring sensitizing EGFR mutations, with receipt or planned receipt of first-line EGFR-TKIs (gefitinib, erlotinib or afatinib); (ii) EGFR mutations with Exon 19 deletion, Exon 21 L858R or another sensitive minor mutation (i.e. Exon 18 G719X); (iii) Eastern Cooperative Oncology Group performance status of 0 to 2. Patients were excluded if they had undergone prior EGFR-TKI treatment with disease progression (PD).

### Plasma ctDNA analysis

Plasma ctDNA was collected at baseline and every 1–2 months. The following events were specifically noted: (i) radiological PD, (ii) clinical PD, (iii) re-biopsy at PD and (iv) treatment change. Plasma ctDNA was analyzed at SRL Laboratory (Tokyo, Japan) using the cobas® EGFR Mutation Test version 2 (v2) to detect sensitizing EGFR mutations and the T790M mutation.

### Re-biopsy and EGFR mutation analysis

When the disease had progressed, tissue re-biopsy was recommended. The EGFR genotypes of re-biopsied materials were analyzed at each hospital using the peptide nucleic acid-locked nucleic acid clamp method or the cobas® EGFR Mutation Test v2.

### Clinical data collection

The case report form (CRF) included clinical information about radiological PD (date, site of PD), clinical PD (date, pattern of PD), survival (date last verified), death (date) and cause of death. Radiological PD was assessed according to the Response Evaluation Criteria in Solid Tumors v1.1 at each institution. Adverse events were assessed according to the Common Terminology Criteria for Adverse Events v4.0.

### Statistical analysis

This study is an observational study to estimate the usefulness of plasma ctDNA monitoring in patients with EGFR mutant NSCLC who received first-line EGFR-TKIs. The primary endpoint was the plasma ctDNA T790M-positivity rate using the cobas® EGFR Mutation Test v2, and secondary endpoints were the best response rate and progression-free survival (PFS) with osimertinib. We defined PFS in this study as from the date of osimertinib initiation to the date of RECIST PD or death. This study used descriptive statistics and the target sample size was set to 120 cases in consideration of the feasibility of the research.

### Ethical considerations

This study protocol was approved by the institutional review board at each participating institution. Declaration of Helsinki ethical standards and local and national regulations were followed. All patients provided written informed consent before participation.

## Results

From September 2016 to March 2017, 122 patients were enrolled in this study. One patient was excluded because he was primarily refractory to first-line EGFR-TKI therapy. EGFR-TKIs were used continuously in 103 patients before enrollment and in 18 patients after enrollment; the first-line EGFR-TKI regimen was gefitinib in 50 patients, erlotinib in 40 patients and afatinib in 31 patients. For more information, please refer to our previous report (7). At the data cut-off, the median follow-up duration was 33.0 months (26.6–33.8).

The final result for the concordance rate of T790M identification between the re-biopsied tissue (*N* = 19) and plasma ctDNA was 21.1% [95% confidence interval (CI): 6.1–45.6] (Table 1).

**Table 1:** Final results of T790M concordance between re-biopsied tissue and plasma

		T790M in plasma		
		Positive	Negative	Total
T790M in tissue	Positive	4 (21.1%)	15 (79.0%)	19
	Negative	5 (16.7%)	25 (83.3%)	30
	Unknown	16 (22.2%)	56 (77.8%)	72
	Total	25 (20.1%)	96 (79.3%)	121

### The best response treated with osimertinib

The best response in all 46 patients treated with osimertinib after a first- or second-generation EGFR-TKIs was 30.4% (95% CI: 17.7–45.8). In the plasma ctDNA T790M-positive group (Liquid-positive group; LP), the response rate was 25.0% (95% CI: 9.8–46.7). There were 14 cases with plasma ctDNA T790M negative/tissue T790M positive (Liquid-negative group; LN), and here, the response rate was 28.6% (95% CI: 8.4–58.1). There was no statistically significant difference between the two groups (*P* = 0.29). There were eight patients in whom both plasma ctDNA and tissue T790M were negative or unknown, and four responded to osimertinib. Three patients in this group discontinued prior EGFR-TKIs due to adverse events without PD and were switched to osimertinib without re-biopsy; two responded (Table 2).

**Table 2:** The best overall response to osimertinib by ctDNA status

ctDNA T790M	Tissue T790M	*N*	RR (%, 95% CI)	DCR (%, 95% CI)
+	+/−	24^*1^	25.0 (9.8–46.7)	66.7 (44.7–84.4)
−	+	14	28.6 (8.4–58.1)	71.4 (41.2–91.6)
−/ukn	−/ukn	8^*2^	50.0 (15.7–84.3)	87.5 (47.4–99.7)
All	All	46	30.4 (17.7–45.8)	71.7 (56.5–84.0)

^*^1: One of the 25 patients with positive plasma ctDNA T790M was excluded from the analysis because osimertinib was not used.

^*^2: This group included three patients who discontinued first- or second-generation EGFR-TKIs due to adverse events and were switched to osimertinib without T790M re-biopsy.

ctDNA, circulating tumor DNA; RR, response rate; DCR, disease control rate; ukn, unknown.

### PFS treated with osimertinib

**Figure 1:**
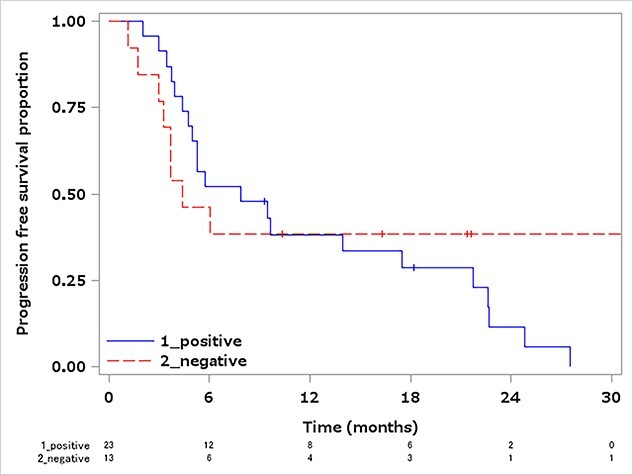
PFS in the ctDNA T790M-positive group and the ctDNA-negative/tissue T790M-positive group, treated with osimertinib. Median PFS in the ctDNA T790M-positive group: 7.9 months (95% CI: 4.7–17.5). Median PFS in the ctDNA-negative/tissue T790M-positive group: 4.4 months (95% CI: 3.0–N.E.). Log-rank test *P* = 0.74. PFS, progression-free survival; ctDNA, circulating tumor DNA; CI, confidential interval; N.E., could not evaluable.

PFS with osimertinib was estimated by the Kaplan–Meier method. The median PFS was 7.9 months (4.7–17.5) in the LP group and 4.4 months (3.0–N.E.) in the LN group, without a statistically significant difference (*P* = 0.74) (Fig. 1).

## Discussion

In the AURA3 study, Papadimitrakopoulou et al. reported that the T790M mutant concordance rate between tissue and liquid biopsy was 51% (110 of 215 samples) using the cobas® EGFR Mutation Test v2 (8). In our study, it was lower, at 21.1% (95% CI: 6.1–45.6%). As heterogeneity is known to occur in NSCLC, it is possible that T790M was not detected in the small re-biopsied samples and appeared at other sites. Papadimitrakopoulou et al. also reported that the detection rate in plasma was related to the baseline size of the tumor target lesion, with a median diameter of 56 mm in cases with detection and 39 mm in those without; the detection rate was also higher in cases with extrathoracic metastatic disease. There are some reports that the frequency of plasma T790M positivity varies depending on the presence of extrathoracic lesions or the site of exacerbation (9–10). Thus, when trying to detect plasma T790M in a single-point test, it is more frequently detected when the tumor target size is larger or there is extrathoracic metastasis. On the other hand, in our study, we monitored plasma T790Mregularly. As a result, plasma T790M might be detected with smaller tumor size and better control of extrathoracic lesions than AURA3, and osimertinib might have been initiated earlier than AURA3.

The best overall response was 25.0% in the LP group and 28.6% in the LN group. In a pooled analysis of the AURA extension and AURA2 trials, the overall response in the tissue T790M-positive group was 66% and in the plasma T790M-negative/tissue-positive group was 70%. The overall response rate was 71% in the AURA3 trial (11). Compared to these reports, the response rate in this study is low. As mentioned above, in our study, plasma ctDNA was measured regularly, which may have led to the initiation of osimertinib while the tumor was still small. Moreover, the timing of the imaging was not consistent due to the clinical setting.

In AURA3, the median PFS of osimertinib for patients who were T790M positive in tissue was 10.1 months (95% CI: 8.3–12.3) and for patients who were also T790M positive in plasma was 8.2 months (95% CI: 6.8–9.7). In our study, the median PFS was 7.9 months, which was similar to the results of the plasma T790M-positive group in AURA3. In our study, there was no statistical difference in survival between the LP group and LN group, despite AURA3 reporting a tendency for plasma T790M-positive patients to have inferior PFS to plasma T790M-negative patients. Detecting resistance at a relatively early timing of tumor progression by repeated tests of plasma T790M, and the initiation of osimertinib could have had a similar effect to that of the plasma T790M-negative group, although its impact on the prognosis is unknown.

This study has several limitations: (i) the small number of cases, particularly the small number where there was a matching T790M mutation detected in both tissue and plasma. Tissue biopsy could, however, not be mandated in daily practice; (ii) this was an observational study and the timing of imaging tests was not well defined, and no radiologic central review was made. These may have affected the response rate and PFS; (iii) PFS also includes a guarantee-time bias/lead-time bias and thus cannot simply be compared with the results of AURA3 or other studies of EGFR-TKIs; (iv) currently, osimertinib is recommended as first-line therapy (12), but other treatment strategies such as ramucirumab plus erlotinib (13) and afatinib followed by sequential osimertinib (14) are being actively studied. In those studies, osimertinib is considered as second-line therapy. In this study, osimertinib was also used as second-line therapy or later. However, in actual practice, osimertinib is often used as first-line therapy, so the clinical relevance of this study might be limited.

## Conclusions

In this study, we regularly monitored plasma T790M. Although the concordance between tissue and plasma ctDNA for T790M mutations was low, the PFS of the plasma ctDNA-positive group was similar to that of the plasma ctDNA-negative tissue T790M-positive group, suggesting its role in clinical decision-making. Relatively smaller tumor volume at detection of plasma ctDNA T790M during monitoring might affect treatment outcomes in a real-world setting.
